# Τhe Nematicidal Potential of Bioactive *Streptomyces* Strains Isolated from Greek Rhizosphere Soils Tested on *Arabidopsis* Plants of Varying Susceptibility to *Meloidogyne* spp.

**DOI:** 10.3390/plants9060699

**Published:** 2020-05-30

**Authors:** Christianna Meidani, Alexandros Savvidis, Evaggelia Lampropoulou, Aggeliki Sagia, Efstathios Katsifas, Nikolaos Monokrousos, Dimitris G. Hatzinikolaou, Amalia D. Karagouni, Eleni Giannoutsou, Ioannis-Dimosthenis S. Adamakis, Nikoletta G. Ntalli

**Affiliations:** 1 Department of Botany, Faculty of Biology, National and Kapodistrian University of Athens, 157 84 Athens, Greece; sbi1200150@gmail.com (C.M.); alsavvi@biol.uoa.gr (A.S.); lampropoulou.evaggelia@gmail.com (E.L.); grad895@edu.biology.uoc.gr (A.S.); skatsi@biol.uoa.gr (E.K.); dhatzini@biol.uoa.gr (D.G.H.); egianno@biol.uoa.gr (E.G.); iadamaki@biol.uoa.gr (I.-D.S.A.); 2 Department of Science and Technology, International Hellenic University, 57001 Thessaloniki, Greece; nmonokrousos@ihu.gr; 3 Department of Pesticides Control and Phytopharmacy, Benaki Phytopathological Institute, 14561 Athens, Greece

**Keywords:** *Arabidopsis thaliana*, growth promotion, *Meloidogyne* spp., katanin mutants, nematicides, *Streptomyces*

## Abstract

A total of 461 indigenous Streptomycetes strains recovered from various Greek rhizosphere habitats were tested for their bioactivity. All isolates were examined for their ability to suppress the growth of 12 specific target microorganisms. Twenty-six were found to exert antimicrobial activity and were screened for potential nematicidal action. *S. monomycini* ATHUBA 220, *S. colombiensis* ATHUBA 438, *S. colombiensis* ATHUBA 431, and *S. youssoufensis* ATHUBA 546 were proved to have a nematicidal effect and thus were further sequenced. Batch culture supernatants and solvent extracts were assessed for paralysis on *Meloidogyne javanica* and *Meloidogyne incognita* second-stage juveniles (J2). The solvent extracts of *S. monomycini* ATHUBA 220 and *S. colombiensis* ATHUBA 438 had the highest paralysis rates, so these Streptomycetes strains were further on tested for nematodes’ biological cycle arrest on two *Arabidopsis thaliana* plants; the wild type (Col-0) and the katanin mutant *fra2*, which is susceptible to *M. incognita*. Interestingly, *S. monomycini* ATHUBA 220 and *S. colombiensis* ATHUBA 438 were able to negatively affect the *M. incognita* biological cycle in Col-0 and *fra2* respectively, and increased growth in Col-0 upon *M. incognita* infection. However, they were ineffective against *M. javanica*. *Fra2* plants were also proved susceptible to *M. javanica* infestation, with a reduced growth upon treatments with the *Streptomyces* strains. The nematicidal action and the plant-growth modulating abilities of the selected Streptomycetes strains are discussed.

## 1. Introduction

The isolation of new bioactive molecules seems at present of high priority for controlling numerous pests that can infect major crops. Efforts towards the above notion have been made; with the Actinomycetes paving the way since they are the source of 10,000 out of 23,000 substances [1]. *Streptomyces*, a large genus belonging to Actinobacteria phylum and Actinomycetales order, comprises of filamentous bacteria that produce most of all known antibiotics, but also secondary metabolites of potential antibacterial, antifungal, antiviral and even nematicidal activity [1,2]. Up to date, 60% or more than known antimicrobial or plant growth-promoting compounds originate from this genus [3].

Of the various plant pathogens, root-knot nematodes (RKN: *Meloidogyne* spp.) can infest several crops, form complexes with soil-borne pathogens [4], and cause annual worldwide crop-yield-loss of about 78 billion US dollars [5]. To date, root-knot nematodes are one of the hardest agricultural pests to control, as numerous agrochemicals have been sent out of the market (91/414/EEC) and the few remaining are easily biodegradable and furnish low efficacy levels under field conditions [6,7]. Currently, some commercial nematicides are based on the avermectins [8,9] representing macrocyclic lactones generated as fermentation products by *Streptomyces avermitilis*. While also, fervenulin isolated from, *Streptomyces* sp. strain CMU-MH021 exhibits a strong nematicidal effect [10].

*Streptomyces* derived biocontrol agents seem to have a dual role since they are not only able to exert a nematicidal action, but also display plant promoting properties [11]. These properties may be the result of their nutrients recycling capacity or their ability to stimulate plant growth hormone production [11,12,13]. *Streptomyces* can also have a drastic effect on the ability of the root to resist against pathogens by activating enzymes or by improving root zone microecology. This was achieved by altering the percentage of plant growth-promoting bacteria against plant-pathogenic ones [14]. It has been recently shown that *Streptomyces galilaeus* strain KPS-C004 suppressed *M. incognita* and promoted plant growth by increasing plant biomass, shoot, and root length in chili host plants [15]. Similarly, *Streptomyces antibioticus* strain M7 culture supernatant/cells/solvent extracts caused increased mortality of *M. incognita* and promoted growth in tomato plants [16]. Similarly, biocontrol experimentation was also conducted for *M. javanica* control [17].

In the past years, the pace of new compound production from actinomycetes has slowed due to the intense isolation of metabolites from various terrestrial sources achieved the previous decades. Furthermore, available nematicides are no longer able to control effectively crop infestations, creating an urgent need to discover new microorganisms that can be used as biocontrol agents or as a source to produce new nematicidal molecules [18]. Since plants shape their root microbiome through root exudate composition (chemotaxis) and nutritional interactions, Greek rhizosphere habitats provide an environment for the growth of various *Streptomyces* species. By interacting with plants, *Streptomyces* may alter the innate plant responses to phytopathogens. Subsequently, it is of great importance to evaluate the potential of various *Streptomyces* strains for possible use as microbial antagonists [18].

In this study, *Streptomyces* strains, isolated from Greek rhizosphere habitats [19], were initially screened to assess their antimicrobial potential against specific microorganisms used as biocontrol markers. The antimicrobial-effective *Streptomyces* strains were further tested for their nematicidal activity against *M. incognita* and *M. javanica*. In specific (1) both culture supernatants and solvent extracts were tested for the paralysis caused at second-stage juveniles (J2) and (2) spore suspensions of nematode-effective *Streptomyces* strains were further evaluated for the parasites’ biological cycle arrest in *Arabidopsis* host plants, of already proven susceptibility to *M. incognita* [20]. Additional assessment of shoot dry weight was made to lay light on the potential plant growth-promoting effect of every application performed. This study is the first report on Greek indigenous rhizosphere isolates of potential use as biocontrol sources against root-knot nematodes either in the form of culture supernatants or solvents extracts. This study is a first step towards the use of Greek *Streptomyces* as a new sustainable approach to the fight against root-knot nematode (*M. incognita* and *M. javanica*) crop invasion without the use of synthetic pesticides.

## 2. Results

### 2.1. Antimicrobial Activity

A total of 461 *Streptomyces* isolates, recovered from various habitats of Greece [19], were examined for their possible inhibitory activity against specific target microorganisms. From all the *Streptomyces* isolates, only 120 exhibited antimicrobial activities against at least one of the target microorganisms tested (data not shown). Twenty-six out of 120 came up with a remarkable antimicrobial activity against fungal or yeast microorganisms (eukaryotic microorganisms) (Table 1).

Three *Streptomyces* isolates, namely ATHUBA 220, ATHUBA 431, and ATHUBA 438 displayed antimicrobial activity against all of the 12 microbial targets. Judging from the diameter of inhibition halo formed, ATHUBA 220 displays intense antimicrobial activity against all microorganisms tested, especially against all fungal and yeast strains as well as against *Lactobacillus fermentum*. As far as ATHUBA 438 is concerned, antimicrobial activity was obvious against several target microorganisms. Isolate ATHUBA 546, reveled antimicrobial activity against six target strains. Its effect against the Gram-positive target strains was two or three times higher (*B. subtilis DSM 10 and L. fermentum ATCC 9338*) than the other 25 isolates. As will be further described, the four *Streptomyces* isolates ATHUBA 220, ATHUBA 431, ATHUBA 438, and ATHUBA 546 showed a nematicidal effect, in addition to also exerting an antimicrobial activity (Table 1, Supplementary Figure S1).

### 2.2. Nematicidal Activity of Streptomycetes Isolates

**Figure 1:**
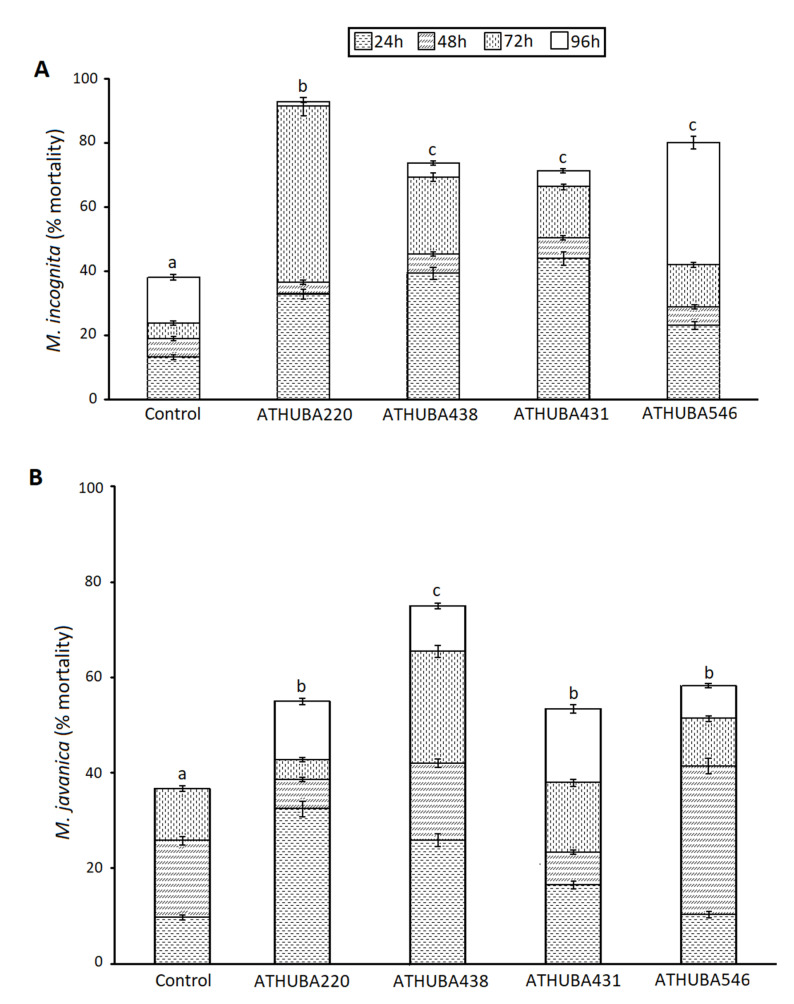
Percentage (mean± standard error) of J2 mortality in *Meloidogyne incognita* (**A**) and *M. javanica* (**B**) after 96 h into batch culture supernatants of different *Streptomyces* strains as depicted. Different letters above columns correspond to statistically significant differences between *Streptomyces* strains (one-way ANOVA, Tukey’s test at *p* < 0.05; for all cases *n* = 15–20).

Twenty-six *Streptomyces* isolates (Table 1) showing antimicrobial activity against eukaryotic cells were in vitro tested for their nematicidal activity to both *M. javanica* and *M. incognita* (data not shown) in the form of culture supernatants. Culture supernatants from only four isolates exhibited a significant increase in the mortality rate of second-stage juveniles J2 of both root-knot nematodes (Figure 1). These were further on accessed and sequenced to be deposited in GeneBank (see material and methods).

In specific, in the presence of culture supernatants of *Streptomyces* strains *S. monomycini* ATHUBA 220 and *S. colombiensis* ATHUBA 438, juveniles of *M. incognita* showed a mortality rate of 92.5% and 73.7% respectively, after 96 h of incubation. The effect of culture supernatants of *Streptomyces* strains *S. colombiensis* ATHUBA 431 and *S. youssoufensis* ATHUBA 546 was also significant when compared to the control. After 96 h of incubation, J2 mortality of 71.2% and 78.9% were observed. Data for these four effective isolates are shown in Figure 1A. Culture supernatants *S. monomycini* ATHUBA 220 and *S. colombiensis* ATHUBA 438 increased *M. javanica* J2s mortality in a percentage of 55% and 75% respectively, after 96 h of incubation. The *M. javanica* J2s treated with culture supernatants of *S. colombiensis* ATHUBA 431 and *S. youssoufensis* ATHUBA 546 exhibited mortality rates of 53.4% and 77.6%, respectively (Figure 1B).

As can be easily observed, the *Streptomyces* isolates culture supernatants lead to earlier paralysis of *M. incognita* than *M. javanica*. In particular, ATHUBA 220 can cause paralysis in 91.1% of the nematodes’ population in just 72 h, whilst the percentage of paralyzed nematodes at the same time at the control was just 33.7%. *M. javanica* nematodes seemed to be affected since 24 h post-experiment establishment, especially in ATHUBA 220 (32.5% mortality in the first 24 h) and ATHUBA 438 (25.9% in the first 24 h). Surprisingly, after 96 h in the culture supernatants, a large proportion of *M. javanica* second-stage juveniles J2 nematodes remained active (Mortality percentage 55% for ATHUBA 220 and 75% for ATHUBA 438). Thus, both nematode species are affected by the specific culture supernatants, but the impact on *M. incognita* vitality is far more severe than that caused in *M. javanica*.

**Figure 2:**
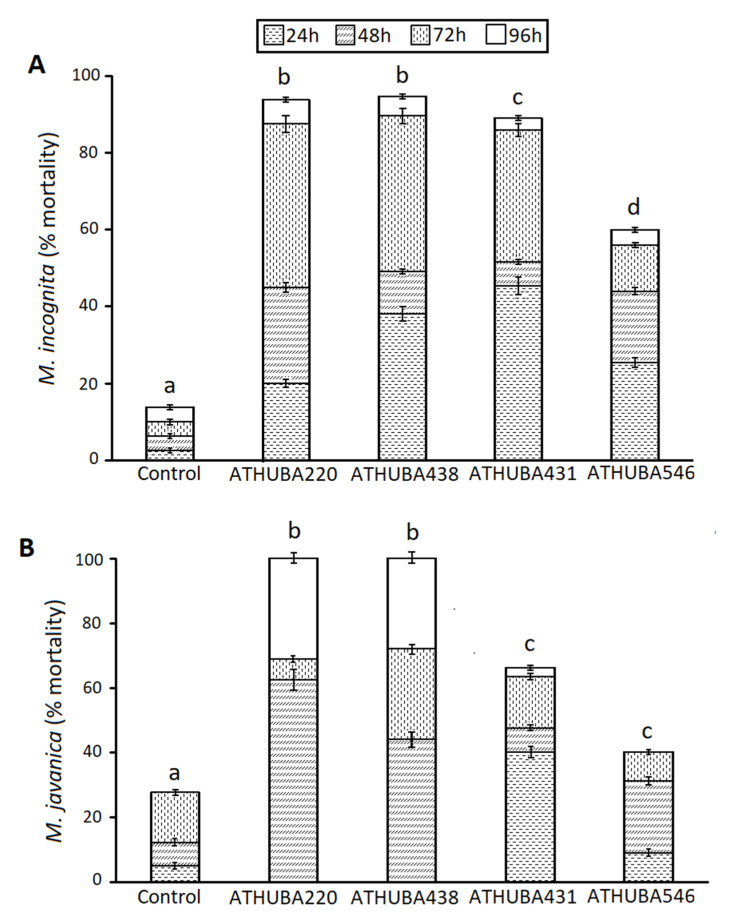
Percentage (mean± standard error) of J2 mortality in *M. incognita* (**A**) and *M. javanica* (**B**) after 96 h of immersion into solvent extracts from different Streptomycetes solid cultures as depicted. The control wells contained 70 μL of J2 s suspension. Different letters above columns correspond to statistically significant differences between treatments (one-way ANOVA, Tukey’s test at *p* < 0.05; for all cases *n* = 15–20).

To confirm nematicidal activity, solvent extracts from each of the above four *Streptomyces* isolates were then tested against J2 s of *M. incognita* and *M. javanica* (Figure 2). In the presence of solvent extracts of *S. monomycini* ATHUBA 220 and *S. colombiensis* ATHUBA 438, juveniles of *M. incognita* showed a mortality rate of 93.8% and 93.7% respectively, after 96 h of incubation. The J2s treated with solvent extracts of *S. colombiensis* ATHUBA 431 and *S. youssoufensis* ATHUBA 546 exhibited mortality rates of 89.1% and 60.0% respectively, after 96 h of incubation. After 48 h of incubation a mortality rate of 51.6% and 44.0% were observed in J2 s of *M. incognita* treated with *S. colombiensis* ATHUBA 431 and *S. youssoufensis* ATHUBA 546, respectively, whereas a mortality rate of 6.3% was observed in control. Data are shown in Figure 2A.

After 48 h of incubation, a mortality rate of 77.5% and 91.2% was observed in J2s of *M. javanica* treated with *S. colombiensis* ATHUBA 431 and *S. youssoufensis* ATHUBA 546, respectively whereas a mortality rate of 27.3% was observed in control. Over a period of 96 h, the mortality of J2s increased to 100% for both examined extracts. Furthermore, J2s incubated 48 h in solvent extracts of *S. colombiensis* ATHUBA 438 and *S. monomycini* ATHUBA 220 exhibited mortality of 44% and 62.5% respectively. Similarly, to the other two strains examined, the percentage of J2s mortality increased up to 100% within 96 h of incubation. Data are shown in Figure 2B. The above data firstly suggest that the solvent extracts used in the experiments are far more drastic against nematodes than the culture supernatant in both *M. incognita* and *M. javanica*. In these experiments, in 48 h, almost half of the nematode population is dead, while at the same time, only a small percentage of the control (6.3%) died.

### 2.3. Nematode Biological Cycle Arrest

**Figure 3:**
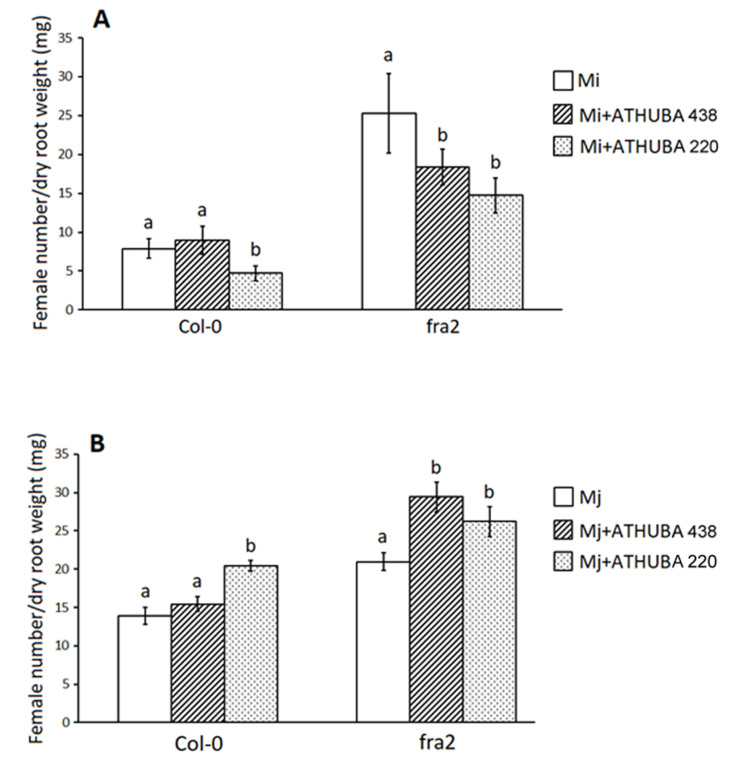
*Arabidopsis thaliana* L. plants (Col-0) and *fra2* katanin mutants root infestation levels expressed as female number/dry root weight (mg) with *M. incognita (*Mi*)*(**A**) and *M. javanica* (Mj) (**B**) 45 Days after application of treatments. The treatments were performed with *Streptomyces monomycini* ATHUBA 220 and *Streptomyces colombiensis* ATHUBA 438 at 10^7^ cells/g of soil. Different letters above columns correspond to statistically significant differences between treatments (one-way ANOVA, Tukey’s test at *p* < 0.05; for all cases *n* = 10).

Roots of *fra2* katanin mutants exhibited more of both nematodes’ females (*M. incognita* and *M. javanica*; Figure 3) per mg of their dry root weight compared to Col-0, indicating that root invasion success was higher in *fra2* mutants (this study; [20]). Treatments performed with spore suspensions of *S. monomycini* ATHUBA 220 could successfully reduce the *M. incognita* burden in both Col-0 and *fra2* (Figure 3A), while ATHUBA 438 was effective and restrained nematode population only in *fra2* mutants. However, that was not the case for *M. javanica*, where neither *S. monomycini* ATHUBA 220 nor *S. colombiensis* ATHUBA 438 treatments seemed to be able to alleviate the *M. javanica* nematode burden (Figure 3B). Thus, it seems that although *S. monomycini* ATHUBA 220 and *S. colombiensis* ATHUBA 438 were effective in the in vitro test, their performance in the biological cycle arrest experimentation was not satisfactory in regard to *M. javanica’s* biological cycle restriction (Figure 3B).

### 2.4. Growth Promotion

**Figure 4:**
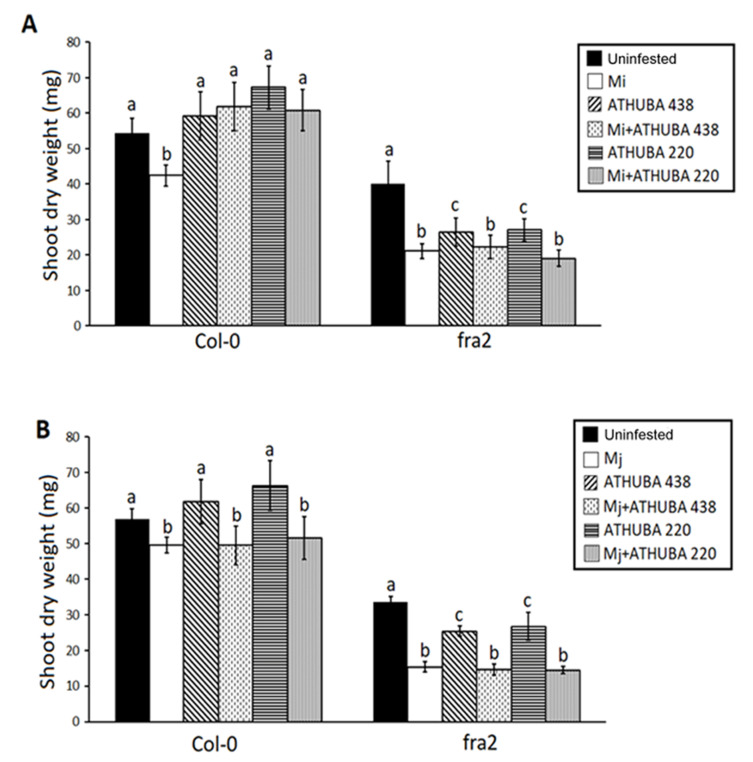
*Arabidopsis thaliana* L. plant (Col-0) and fra2 katanin mutants shoot dry weight (mg), of control (uninfested), infested with either *M. incognita* (**A**) or *M. javanica* (Mj) (**B**) or treated with *Streptomyces monomycini* ATHUBA 220 and *Streptomyces colombiensis* ATHUBA 438 spore suspensions at 10^7^ cells/g of soil (prior Mi/Mj infection or not). 45 days after application of the depicted treatments/infections. Different letters above columns correspond to statistically significant differences between treatments (one-way ANOVA, Tukey’s test at *p* < 0.05; for all cases n = 10).

Nematode burden caused a significant decrease in shoot dry weight in both Col-0 plants and *fra2* katanin mutants (Figure 4). This decrease seemed to be alleviated in *M. incognita* infected Col-0 plants after treatment with both spore suspension *S. monomycini* ATHUBA 220 and *S. colombiensis* ATHUBA 438 prior to *M. incognita* infection (Figure 4A). However, this was not the case in *fra2 M. incognita* infected plants, where *S. monomycini* ATHUBA 220 and *S. colombiensis* ATHUBA 438 treatment failed to alleviate the nematode effects in growth (Figure 4A). The *M. janavica*-caused dry weight decrease did not seem to be relieved by *S. monomycini* ATHUBA 220 and *S. colombiensis* ATHUBA 438 application, neither in Col-0 nor *fra2* plants (Figure 4B). Moreover, treatments with *S. monomycini* ATHUBA 220 and *S. colombiensis* ATHUBA 438 seemed to stress *fra2* plants and caused a reduction in shoot dry mass (Figure 4A,B).

## 3. Discussion

The Greek geomorphological and climate conditions result in soil reservoirs of high taxonomic and functional diversity within the resident *Streptomyces* populations, providing a rich pool of *Streptomyces* strains of potential biotechnological value [21,22,23,24,25,26]. In line with the above notion, in the present work, 26 out of 461 *Streptomyces* isolates, originating from different habitats within the Greek territory [19], showed inhibitory action against 12 target microorganisms (Table 1). Out of the 26 isolates, 3 exhibited a significant inhibitory action against all of the 12 target microorganisms tested (Table 1; Supplementary Figure S1), namely, *S. monomycini* ATHUBA 220, *S. colombiensis* ATHUBA 438, and *S. colombiensis* ATHUBA 431; thus, extending the list of *Streptomyces* spp. isolates with the potential ability to generate a plethora of secondary metabolites that could be used in both medicine and agriculture [27,28]. Only recently *Streptomyces spp.* strains have been considered as prospective biocontrol agents in agriculture. Indeed, their ability to produce drastic biomolecules may be used to control plant pathogenic bacteria and fungi [29]. Thus, the assessment of the isolated *Streptomyces* strains for their capacity to control the root-knot nematodes, as one of the most damaging agricultural pests attacking a wide range of crops, was an important step in their characterization [1,30]. Out of the 26 *Streptomyces* isolates screened for nematicidal activity, the 3 which exhibited an antimicrobial activity against all 12 target microorganisms plus *S. youssoufensis* ATHUBA 546 had a nematicidal effect (Figure 1 and Figure 2).

In 1979, Sawhney and Webster (1979) [31] evaluated in vivo the effect of actinomycin D—a chemical derived from *Streptomyces* sp.—against *M. incognita,* and observed no gall formation in treated tomato plants. Since then, research has been conducted regarding the nematicidal potential of various *Streptomyces* strains [30]. Several *Streptomyces* strains have been characterized as able to produce bioactive metabolites that can inhibit the biological cycle or even kill nematodes [32]. Moreover, *Streptomyces* spp. can be combined with other nematode control measures in the frame of integrated crop management. In particular Jin et al. (2017) [33] showed that the reduction rates of *Meloidogyne incognita* caused by the combined application of *Streptomyces rubrogriseus* HDZ-9-47 with biofumigation were higher than those of HDZ-9-47 and cabbage residue biofumigation used alone or fosthiazate treatment, and tomato yield also increased [33]. As far as we are aware, this is the first report of Greek indigenous *Streptomyces* strains exhibiting nematicidal capabilities. *S. colombiensis* ATHUBA 431, *S. colombiensis* ATHUBA 438, *S. youssoufensis* ATHUBA 546, and *S. monomycini* ATHUBA 220 showed in in vitro experiments a nematicidal effect (Figure 1 and Figure 2). Solvent extracts of *S. colombiensis* ATHUBA 438 and *S. monomycini* ATHUBA 220 showed an increased nematicidal activity reaching for *M. javanica* 100% and 93.8% respectively and 93.7% for *M. incognita,* after 96 h of incubation. Therefore, those two *Streptomyces* strains were further on tested on *M. incognita* and *M. javanica* infestation of wild type *Arabidopsis thaliana* L. plants (Col-0) and the *fra2* katanin mutant which is susceptible to *M. incognita* [20]. While spore suspensions of *S. monomycini* ATHUBA 220 and *S. colombiensis* ATHUBA 438 could alleviate the nematode burden of *M. incognita* in Col-0 and *fra2* (Figure 3A) respectively, to our surprise, this was not the case for *M. javanica*, where *S. colombiensis* ATHUBA 438 and *S. monomycini* ATHUBA 220 were ineffective (Figure 3B). Additionally, it has previously been demonstrated that *M. javanica* is more difficult to control than *M. incognita* [34,35].

*Streptomyces* spp. are considered an important group of soil bacteria, multifunctionally beneficial, enhancing plant and soil health under nematode stress and helping in improving the microbial community structure [36]. Although *Streptomyces* species have the ample capacity to produce plant growth-promoting substances, secondary metabolites (such as antibiotics), enzymes and phytohormones, only a handful of studies deal with the usefulness of the rhizospheric *Streptomyces* spp. in plant growth [37,38,39]. For example, the commercialized *Streptomyces lydicus* WYEC108 strain, as a root-colonizing actinomycete has five properties that either suppress fungal pathogens or enhance plant growth: (1) rhizosphere competent; (2) antibiosis; (3) mycoparasitism; (4) cellulose and chitinase production; and (5) siderophore production [40]. Moreover, *Streptomyces galilaeus* strain KPS-C004 suppressed up to 58% of root-knot disease of chili caused by *Meloidogyne incognita* in a greenhouse experiment, promoted plant growth by increasing biomass and plant nutrients; and did not affect soil bacterial community [16]. Likewise, *S. hydrogenans* strain DH16 and its metabolites were proven to be safe nematicides against *M. incognita* and were characterized by plant growth-promoting properties [41].

In accordance, the two native actinomyces isolates originating from Greek soils (*S. colombiensis* ATHUBA 438 and *S. monomycini* ATHUBA 220) are a good source of nematicidal agents but also seem to promote plant growth. In Col-0 plants treated with either *S. monomycini* ATHUBA 220 or *S. colombiensis* ATHUBA 438, growth promotion under *M. incognita* infestation has been observed (Figure 4). Interestingly, when *fra2* plants were treated with these two streptomyces isolates, the results differed. More particularly, neither of the two *Streptomyces* strains could reverse the infestation-derived growth defect; both also led to a growth reduction of the nematode uninfected *fra2* plants (Figure 4). *Streptomyces* species, especially those who are classified as plant pathogenous, can produce enzymes which affect cellulose biosynthesis (i.e., thaxtomin a) [42]. Moreover, the nonpathogenous strains can produce cellulose enzymes that affect plant cell wall abilities [36]. *Fra2* mutants produce very low cellulose [43]; therefore, the cellulosic enzymes of the Streptomycetes strains could affect the already deteriorated cell walls of the mutants and thus enhance the observed growth decrease (Figure 4).

Captivatingly, *S. colombiensis* is commercially used as a myocide against powdery mildews, grey mold, and brown patch [44]. In contrast, *S. monomycini* strain C 801 alleviated water stress, salt stress in peppermint [45] and wheat [46] cultivars respectively, and suppressed *Phytophthora drechsleri* in cucumber plants [47]. Our research expands the bioactivity of *S. colombiensis* and *S. monomycini* species with the characterization of ATHUBA 431, ATHUBA 438 and ATHUBA 220 strains as sources of potential nematicidal agents, and in addition, an *S. youssoufensis* strain ATHUBA 546 is also described as such. Further investigation of the nature of the specific nematicidal substances produced by the *S. colombiensis* ATHUBA 431, *S. youssoufensis* ATHUBA 546, *S. colombiensis* ATHUBA 438 and *S. monomycini* ATHUBA 220 is required, and will be dealt with in the near future.

## 4. Materials and Methods

### 4.1. Microbial Strain Isolation and Nematode Rearing

The *Streptomyces* strains used in this study were isolated from soil samples of three different areas of Greece (Table 2). 461 was the total number of the *Streptomyces* strains used in this study. The 344 strains were isolated in the frame of a study performed previously [19] and 117 from the rhizosphere of the plant’s *Veronica oetaea* and *Olea europaea*. The analytical sampling procedure followed is described in [19]. The target nematode populations of *M. incognita* and *M. javanica*, were reared on tomato plants (*Solanum lycopersicum* Mill.) cv. Belladonna. Second stage juveniles J2, hatched every 2 days were extracted from egg masses according to Hussey and Barker (1973) [48] from 60 days (d) nematode-infested roots and were used for the bioassays.

### 4.2. Streptomyces Growth Media and Growth Conditions

All media used in this study are described in [49]. Specifically, the medium used for the production of spores of *Streptomyces* isolates was tryptone, glycerol, salts agar—TGSA containing (g L^−1^): glycerol 12.5, tryptone 5.0, K_2_HPO_4_ 1.0, MgSO_4_ 7H_2_O 0.5 and 1 mL/L of a salt solution of Fe_2_ (SO_4_)_3_ 1.0 Μ, CuSO_4_ 0.1 Μ, ZnSO_4_ 0.1 Μ and MnSO_4_ 0.1 Μ and the pH was adjusted at 7.8. Thereafter, 15.0 g of agar was added before the sterilization. Media used for the growth of the target microorganisms were prepared as follows: For Gram-negative and Gram-positive bacteria, nutrient agar—NA containing (g L^−1^) nutrient broth 13.0 and agar 10.0 was used. For the estimation of antimicrobial activity against the Gram-positive strain *Lactobacillus fermentum* ATCC 9338, the Man, Rogosa & Sharpe Agar—MRS was used. The MRS Agar contained (g L^−1^) MRS (Sharlau Chemie S.A.) 52.0 and agar 10.0.

Medium used for the antimicrobial estimation against the yeasts target strains was malt extract agar—MEA containing (g L^−1^) malt extract broth (Sharlau, Chemie S.A.) 20.0 and agar 10.0. Medium used for the antimicrobial estimation against the fungal target strains was potato dextrose agar—PDA containing (g L^−1^) potato peptone 4.0, glucose 20.0, and agar 10.0.

All *Streptomyces* isolates were incubated in TGSA plates for spore production for 7 days at 30 °C. The results shown in Table 1 and Figure S1 are the mean of three independent experiments.

The medium TGSA without the agar addition, named TGSB, was used for the batch cultivation of the *Streptomyces* isolates for DNA extraction.

### 4.3. Preliminary Screening for Antimicrobial Activity

In this study *Streptomyces* isolates were examined for possible inhibitory activity against various target microorganisms. Following the protocol described by Raghava et al., 2017 [50] three Gram positive bacteria (*Bacillus subtilis* DSM 10, *Micrococcus luteus* DSM 1790, *Lactobacillus fermentum* ATCC 9338); four Gram negative bacteria (*Escherichia coli* NEB DH 5a, *Pseudomonas aeruginosa* ATCC 15442, *Pseudomonas fluorescens* DSM 50090, *Acinetobacter radioresistens* DSM 6976); two yeasts (*Saccharomyces cerevisiae* DSM 70449, *Cyberlindera saturnus Minter* ATHUM 2576) and three filamentous fungi (*Aspergillus niger* DSM 1957, *Aspergillus nidulans* LA_1_, *Fusarium oxysporum* DSM 62059), were selected. Then, 25 μL glycerol suspension of each *Streptomyces* isolate was inoculated on TGSA plates in triplicates. Plates were incubated for 48 h at 30 °C. Thereafter, the *Streptomyces* colonies were irradiated for 20 min under UV lamp.

Every target microorganism was prepared as follows: 100 mL of the appropriate for each microorganism agar medium was prepared and sterilized in Duran bottles of 250 mL. NA was prepared for all Gram-positive and Gram-negative bacteria (except L. *fermentum* ATCC 9338 strain); MRS agar for the L. *fermentum* ATCC 9338, MEA for yeasts and PDA for the fungal strains. After the sterilization, and when the temperature of the agar medium decreased at 50 °C, 0.5 mL of a glycerol suspension of the microbial target strain was introduced in the agar medium and stirred for 60 s. Thereafter, 20 mL of each agar medium were placed above the irradiated *Streptomyces* colonies. Plates containing the bacteria and yeasts target strains were incubated at 30 °C for 24 h and the plates containing the fungal strains were incubated for 48 h. Antimicrobial activity was determined by measuring the diameter of the inhibition halo formed around the *Streptomyces* colonies. [51].

### 4.4. Screening of Streptomyces Isolates for Nematicidal Activity

For culture supernatant paralysis bioassay, each isolate of *Streptomyces* was grown in 100 mL conical flasks containing 20 mL *TGSB* medium. The medium was inoculated with 1 × 10^9^ of spore suspension of each isolate. All batch cultures were grown at 180 rpm and 30 °C for 7 days. The cultures were then centrifuged at 4000 rpm for 15 min and the supernatants were examined for possible inhibitory activity against root-knot nematodes. Supernatants were tested for paralysis against second-stage juveniles (J2) of *M. javanica* and *M. incognita*. A 70 microliters J2 suspension, containing 10–20 J2, was transferred into each well of a 96-well culture plate containing 70 microliters of *Streptomyces* supernatants. As negative control, 70 microliters of a J2 suspension dissolved in 70 microliters culture nutrient was used. Three replicates were performed for each supernatant/treatment. The plate was incubated at room temperature and dead juveniles were counted under a Zeiss inverted microscope, every 12 h, for 96 h period. Motility recovery was assessed by transferring J2 in tap water after the last assessment (96 h) and observing again after 1 day. Since no differences were evident, J2 were considered dead and the results presented herein are those calculated before rinsing. The immobile, malformed, or motionless juveniles when probed with a needle were considered as dead. All experiments were carried out in triplicate. The plates were covered to prevent evaporation and were maintained in continuous light at 25 °C.

For solvent extract paralysis bioassay, procedures were established as described by [52] with few modifications. *S. monomycini* ATHUBA 220, *S. colombiensis* ATHUBA 438, *S. colombiensis* ATHUBA 431 and *S. youssoufensis* ATHUBA 546 were streaked at straight lines onto TGSA plates and grown for 7 days at 30 °C. For the confirmation of bioactivity, one inoculated plate was assessed as described in Section 4.3. Agar from the plates (without any colonies of microorganism) was harvested, disrupted through a 50 mL syringe and frozen overnight at −20 °C. A water-based extract was produced by squeezing the thawed slurry through muslin cheesecloth and filtering the resultant liquid through Whatman filter paper. A concentrated extract was obtained by freeze drying the filtered extract in a Crist Alpha 1–4 freeze dryer and resuspending the lyophilized material in 3 mL of sterile water. Acetone-based extract was produced by squeezing the mixture through muslin cheesecloth, filtering it and removal of acetone using a vacuum rotary evaporator to generate a concentrated extract comprising the residual water present in the spent agar–cell slurry. The final extract, named the solvent extract, was established after filtering the concentrated extract through 0.2 μm pore size sterile filters. Experimental treatments were laid in a 96-well culture plate for culture supernatant paralysis bioassay, with the same experimental design and controls. Again, 70 microliters J2 suspension, containing 10–20 J2, was transferred into each well of a 96-well culture plate containing 70 microliters of *Streptomyces* culture solvent extract. Solvent extracts derived from agar without any colonies of microorganism were used as controls.

### 4.5. DNA Amplification and Sequencing of 16S rDNA Gene

The selected *Streptomyces* isolates were harvested from 1 mL overnight culture in TGSB medium at 30 °C and 200 rev min^−1^. DNA was extracted using the CTAB (Cetyl trimethylammonium bromide) protocol described in the ISO 21571:2005 and was finally diluted in 100 μL distilled water and preserved at −80 °C. The DNA concentration was determined by spectrophotometer and adjusted to 40 ng μl^−1^ for PCR reaction. The region of its 16S rRNA was amplified by PCR using primers R1492 (5’-TAC-GGY-TAC-CTT-GTT-ACG-ACT-T-3’) and pA (5’-AGA-GTT-TGA-TCC-TGG-CTC-AG-3’). The conditions for PCR reaction were as follows: denaturation of target DNA at 95 °C for 2 min followed by 35 cycles at 95 °C for 30 sec, primer annealing at 53 °C for 30 sec, primer extension at 72 °C for 2 min and final extension at 72 °C for 5 min. Thereafter, 16S rRNA PCR products were extracted from 0.8% (*w/v*) agarose gel using the NucleoSpin Gel and PCR Clean-up kit (REF 730609.10, Macherey-Nagel GmbH & Co., Düren, Germany) following the manufacturer’s protocol. Sequence data were obtained by capillary electrophoresis (VBC-Biotech Service GmbH, Eurofins, Vienna). Each product was sequenced in one direction. The size of each sequence was approximately 900 bp. Similarity searches of the GenBank database were performed with BLAST [53]. Sequences retrieved from this study have GenBank codes MT000152, MN860152, MN854384, MN853681.

### 4.6. Biological Cycle Arrest

The impact of the two-best performing actinomyces in paralysis experiments were further used in pot bioassays. In particular, we studied the effect of *S. monomycini* ATHUBA 220 and *S. colombiensis* ATHUBA 438 spore suspension application, prior to *M. incognita* and *M. javanica* infestation in wild type *Arabidopsis thaliana L.* plants (ecotype Columbia; Col-0) and *fragile fiber 2* (*fra2*) katanin mutant (*fra2* is already proven susceptible to *M. incognita* [20]). *Streptomyces* spore suspensions were added in soil where *Arabidopsis* plants were established 15 days before the artificial inoculation with the nematodes, to impede root penetration by *M. javanica* and *M. incognita* via parasitism or secondary metabolites’ production. 15 days are considered by the literature as a sufficient period of time for *Streptomyces* isolates to grow and adapt to their new environment, and/or produce toxic compounds before the root knot nematode penetrate the plant cells [54].

Seeds of *A. thaliana* (as previously reported; [55] (purchased from the NASC European Arabidopsis Stock Centre, Nottingham, UK) were surface-sterilized with a 30% (*v*/*v*) bleach solution and kept at 4 °C for 48 h. Subsequently, the seeds were transferred in sterilized soil (Postground P, Klasmann-Deilmann, Geeste, Germany) and left to germinate and grow in a growth chamber under a constant 16 h day/8 h night regime at an ambient temperature of 21 ± 1 °C, with light intensity set at 120 μmol m^−2^·s^−1^, in 64 mL pots. The test concentration for both *Streptomyces monomycini* ATHUBA 220 and *Streptomyces colombiensis* ATHUBA 438, on *M. javanica* and *M. incognita* was 10^7^ cells/g of soil. *Streptomyces* spore suspensions were added to the soil of 12-day-old plants and 15 days later the soil was infested with *M. javanica* and *M. incognita* suspension of 20 J2/g soil. 47 days post nematode inoculation (dpi) the plants were uprooted, and the roots were gently washed under water to remove soil debris. The plants were first used to assess plant growth parameters and then were subjected to nematodes counts. The aerial part of the plant was separated from roots, the roots were transferred into 10 mL falcons and they were stained with acid fuchsin [56]. Dry shoot mass and the number of female nematodes per mg of root dry mass were estimated 47 days post nematodes’ inoculation. The females inside the roots were counted at 10× magnification under uniform illumination by transparent light. Nematode burden was expressed as number of females per mg dry root weight. The experiment was performed twice, and the treatments were always arranged in a completely randomized design with five replicates.

### 4.7. Shoot Dry Weight Recording

Ten shoots from Col-0 and *fra2* plants after various treatments were excised and were further on assessed as stated in [57]. In short, samples were dried in an oven at 70 °C for 72 h when constant weight was reached. Dried shoots and roots were weighed on a scale (±0.001 g).

### 4.8. Statistical Analysis

Considering nematicidal bioassays, ANOVAs indicated no significant treatment between runs of experiment, and thus, means were averaged over experiments. No data transformation took place judging from the normality and homogeneity of variance tests. Statistical analysis was performed using SPSS 20 (IBM, Armonk, NY, USA). Both ANOVA and Tukey’s tests were set at *p* ≤ 0.05.

## 5. Conclusions

This study is the first report demonstrating a nematicidal potential of Greek soil-indigenous *Streptomyces* strains against *M. incognita* and *M. javanica.* The results of *in vitro* and *in vivo* studies indicate that culture supernatants, cell solvent extracts and spore suspensions have a potential to control nematode infestation and to reduce its detrimental effects on crop production. Although *M. javanica* appeared resilient under soil conditions towards *Streptomyces* strains, the described Greek *Streptomyces* isolates might be a source for the production of safe biopesticides to control *M. incognita* and *M. javanica* as well as effective biofertilizers to promote plant growth upon root-knot nematode infection.

## Figures and Tables

**Table 1:** Diameter of inhibition halo (mm ± standard error) formed around each of the 26 *Streptomyces* isolates, tested against the 12 target microorganisms.

*Streptomyces* sp.	*Aspergillus niger* *DSM 1957*	*Aspergillus nidulans* *LA_1_*	*Fusarium oxysporum DSM 62059*	*Saccharomyces cerevisiae* *DSM 70449*	*Cyberlindera* *saturnus Minter ATHUM 2576*	*Escherichia* *coli NEB DH 5a*	*Pseudomonas aeruginosa* *ATCC 15442*	*Pseudomonas fluorescens* *DSM 50090*	*Acinetobacter radioresistens DSM 6976*	*Bacillus subtilis DSM 10*	*Micrococcus luteus DSM 1790*	*Lactobacillus fermentum ATCC 9338*
*ATHUBa* 127	4.8 ± 0.2	0	0	7.3 ± 0.5	12.2 ± 1.0	0	0	16.0 ± 1.1	2.3 ± 0.2	3.6 ± 0.4	13.0 ± 0.6	4.0 ± 0.3
*ATHUBa* 220	6.2 ± 0.4	9.0 ± 0.9	12.2 ± 0.8	16.8 ± 1.3	13.7 ± 0.9	2.2 ± 0.2	3.2 ± 0.2	2.2 ± 0.2	12.3 ± 0.6	3.6 ± 0.3	2.2 ± 0.1	20.3 ± 1.8
*ATHUBa* 309	2.0 ± 0.1	0	0	7.3 ± 0.5	23.0 ± 1.1	0	0	2.6 ± 0.1	0	0	4.0 ± 0.3	4.0 ± 0.4
*ATHUBa* 371	0	0	2.2 ± 0.1	4.0 ± 0.2	0	2.3 ± 0.1	0	6.2 ± 0.5	12.3 ± 0.9	6.2 ± 0.4	2.3 ± 0.1	2.6 ± 0.2
*ATHUBa* 393	0	0	2.6 ± 0.2	4.0 ± 0.3	0	4.0 ± 0.4	0	4.0 ± 0.4	6.3 ± 0.5	6.2 ± 0.4	2.6 ± 0.2	4.8 ± 0.3
*ATHUBa* 405	0	0	3.6 ± 0.3	6.2 ± 0.4	0	4.0 ± 0.3	0	1.2 ± 0.1	5.8 ± 0.4	4.0 ± 0.4	6.2 ± 0.5	4.0 ± 0.2
*ATHUBa* 421	0	0	2.9 ± 0.2	4.0 ± 0.4	0	2.6 ± 0.2	0	4.8 ± 0.2	2.6 ± 0.2	3.6 ± 0.3	0	2.6 ± 0.2
*ATHUBa* 431	4.0 ± 0.4	2.6 ± 0.2	8.4 ± 0.6	9.0± 0.9	12.2 ± 0.7	6.2 ± 0.5	3.6 ± 0.3	12.2 ± 0.6	25.0 ± 1.7	9.0 ± 0.6	20.2 ± 1.4	6.2 ± 0.5
*ATHUBa* 438	2.2 ± 0.1	1.9 ± 0.1	6.2 ± 0.6	6.8 ± 0.7	12.2 ± 0.8	7.8 ± 0.6	6.3 ± 0.4	10.9 ± 0.9	25.0 ± 1.5	5.3 ± 0.4	19.4 ± 1.3	6.2 ± 0.6
*ATHUBa* 501	6.2 ± 0.4	4.0 ± 0.3	2.6 ± 0.0	1.4 ± 0.1	2.6 ± 0.2	0	0	2.3 ± 0.1	6.8 ± 0.5	5.3 ± 0.4	9.0 ± 0.5	16.0 ± 1.1
*ATHUBa* 526	2.2 ± 0.2	2.2 ± 0.1	2.0 ± 0.2	2.6 ± 0.2	1.7 ± 0.2	0	0	2.0 ± 0.1	4.0 ± 0.4	0	1.2 ± 0.1	0
*ATHUBa* 527	2.2 ± 0.2	2.2 ± 0.2	1.7 ± 0.2	2.9 ± 0.2	3.2 ± 0.3	0	0	2.6 ± 0.2	2.9 ± 0.3	0	3.2 ± 0.3	0
*ATHUBa* 531	3.6 ± 0.4	2.3 ± 0.2	1.4 ± 0.1	2.9 ± 0.2	1.9 ± 0.2	0	0	4.0 ± 0.2	4.0 ± 0.3	1.4 ± 0.1	2.6 ± 0.2	0
*ATHUBa* 546	0	12.2 ± 1.0	0	4.0 ± 0.2	0	0	0	4.0 ± 0.3	0	26.0 ± 1.6	9.0 ± 0.5	49.0 ± 3.2
*ATHUBa* 576	0	4.0 ± 0.2	0	2.0 ± 0.2	2.6 ± 0.2	0	0	5.8 ± 0.6	2.6 ± 0.2	2.6 ± 0.2	6.2 ± 0.6	2.3 ± 0.1
*ATHUBa* 133	3.6 ± 0.2	0	0	7.3 ± 0.7	12.2 ± 0.8	0	0	0	0	0	0	0
*ATHUBa* 178	0	0	0	9.0 ± 0.9	12.9 ± 0.9	0	0	0	0	0	1.4 ± 0.1	0
*ATHUBa* 181	0	0	2.0 ± 0.1	9.0 ± 0.8	12.2 ± 0.7	0	0	0	0	0	1.0 ± 0.2	0
*ATHUBa* 183	2.2 ± 0.1	0	3.6 ± 0.4	10.2 ± 1.0	12.2 ± 0.6	0	0	0	0	0	1.2 ± 0.1	0
*ATHUBa* 187	1.4 ± 0.1	0	1.9 ± 0.1	9.0 ± 0.6	12.2 ± 1.1	0	0	0	0	0	0	0
*ATHUBa* 200	6.8 ± 0.5	0	0	16.0 ± 0.9	16.0 ± 1.1	0	0	0	0	0	0	0
*ATHUBa* 201	6.2± 0.5	0	0	15.2 ± 1.1	12.3 ± 0.7	0	0	0	0	0	0	0
*ATHUBa* 262	1.7 ± 0.1	0	0	6.8 ± 0.7	10.2 ± 1.0	0	0	0	0	0	0	1.4 ± 0.1
*ATHUBa* 307	0	0	0	6.2 ± 0.5	12.2 ± 0.7	0	0	0	0	0	1.2 ± 0.1	0
*ATHUBa* 309	1.9 ± 0.1	0	0	7.3 ± 0.7	23.0 ± 1.4	0	0	2.6 ± 0.1	0	0	4.0 ± 0.3	4.1 ± 0.2
*ATHUBa* 358	0	0	4.0 ± 0.3	4.8 ± 0.3	9.6 ± 0.7	0	0	0	0	0	0	0

**Table 2:** Description of the sites where the nematicidal isolates were recovered from.

	Ref.	Type of Ecosystem
*S. colombiensis* **ATHUBA 431**	[19]	Rhizosphere of *Pinus brutia*
*S. colombiensis* **ATHUBA 438**	[19]	Rhizosphere of *Pinus brutia*
*S. youssoufensis* **ATHUBA 546**	[Present work]	Rhizosphere of *Olea europea*
*S. monomycini* **ATHUBA 220**	[19]	Rhizosphere of evergreen woody shrubs

## Supplementary Materials

The following are available online at https://www.mdpi.com/2223-7747/9/6/699/s1, Figure S1: The inhibition halo diameter (mm± standard error) of the four *Streptomyces* isolates (ATHUBA 220, ATHUBA 431, ATHUBA 438 and ATHUBA 546) displaying nematicidal activity.
