# Supplementary material for: Τhe Nematicidal Potential of Bioactive Streptomyces Strains Isolated from Greek Rhizosphere Soils Tested on Arabidopsis Plants of Varying Susceptibility to Meloidogyne spp
Source: Plants (Basel). 2020 May 30;9(6):699. doi: 10.3390/plants9060699 (PMC7355556; doi:10.3390/plants9060699)
Supplement: Supplementary file 1 [file plants-09-00699-s001.pdf]

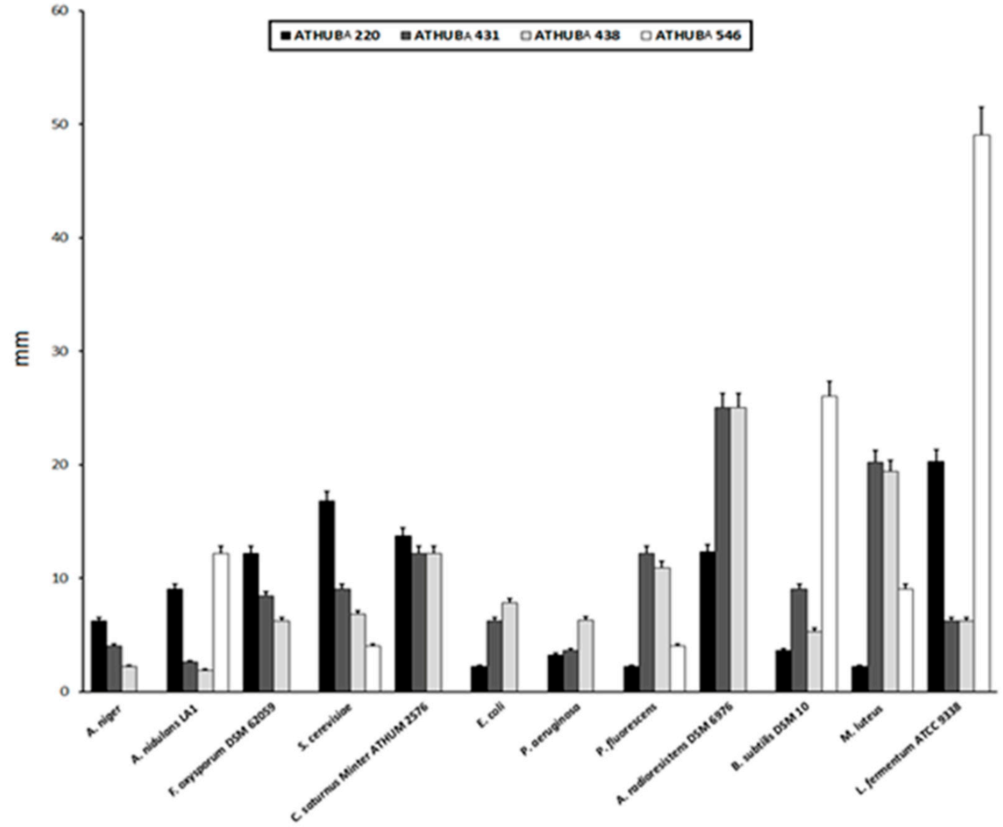

Figure S1. The inhibition halo diameter (mm± standard error) of the four *Streptomyces* isolates (ATHUBA 220, ATHUBA 431, ATHUBA 438 and ATHUBA 546) displaying nematicidal activity.
